# The complex burden of determining prevalence rates of mild cognitive impairment: A systematic review

**DOI:** 10.3389/fpsyt.2022.960648

**Published:** 2022-09-23

**Authors:** Maria Casagrande, Giulia Marselli, Francesca Agostini, Giuseppe Forte, Francesca Favieri, Angela Guarino

**Affiliations:** ^1^Department of Dynamic and Clinical Psychology and Health Studies, “Sapienza” University of Rome, Rome, Italy; ^2^Department of Psychology, “Sapienza” University of Rome, Rome, Italy; ^3^Body and Action Laboratory, Istituto di Ricovero e Cura a Carattere Scientifico (IRCCS) Santa Lucia Foundation, Rome, Italy

**Keywords:** mild cognitive impairment (MCI), prevalence, epidemiology, systematic review, MCI diagnostic criteria

## Abstract

Mild cognitive impairment (MCI) is a syndrome characterized by a decline in cognitive performance greater than expected for an individual's age and education level, but that does not interfere much with daily life activities. Establishing the prevalence of MCI is very important for both clinical and research fields. In fact, in a certain percentage of cases, MCI represents a prodromal condition for the development of dementia. Accordingly, it is important to identify the characteristics of MCI that allow us to predict the development of dementia. Also, initial detection of cognitive decline can allow the early implementation of prevention programs aimed at counteracting or slowing it down. To this end, it is important to have a clear picture of the prevalence of MCI and, consequently, of the diagnostic criteria used. According to these issues, this systematic review aims to analyze MCI prevalence, exploring the methods for diagnosing MCI that determine its prevalence. The review process was conducted according to the PRISMA statement. Three thousand one hundred twenty-one international articles were screened, and sixty-six were retained. In these studies, which involved 157,035 subjects, the prevalence of MCI ranged from 1.2 to 87%. The review results showed a large heterogeneity among studies due to differences in the subjects' recruitment, the diagnostic criteria, the assessed cognitive domains, and other methodological aspects that account for a higher range of MCI prevalence. This large heterogeneity prevents drawing any firm conclusion about the prevalence of MCI.

## Introduction

Age is the biggest risk factor for the development of cognitive impairment. However, many other aspects could be associated with worsening cognitive functions, such as socioeconomic status, genetics, education, environmental exposure, and other comorbidities (1, 2).

In this regard, mild cognitive impairment (MCI) is a syndrome defined by a decline in cognitive performance greater than expected for an individual's age and education level, but that does not interfere much with daily life activities (1). It is distinct from dementia, characterized by more severe cognitive deficits that affect daily functions. According to Petersen (3), MCI represents an intermediate stage of a continuum that ranges from normal cognitive functioning to dementia (i.e., Alzheimer's disease). Various categories of MCI are described, including benign senescent forgetfulness (4), cognitive impairment, and no dementia [CIND; (5)]. The common characteristic is a cognitive deterioration that is insufficient to diagnose dementia (6).

There is not a consensus on the criteria for diagnosing MCI. At first, MCI patients were described as having only a memory impairment (7). However, more recently, the concept of MCI has been expanded to include other types of cognitive impairments beyond memory. Petersen (8) hypothesized a categorization in multiple subtypes based on the nature and number of impaired domains. According to this hypothesis, it is possible to distinguish: (a) amnestic MCI single domain, characterized by an impairment only in the memory domain (aMCIsd); (b) amnestic MCI multiple domains marked by impairments in memory and other neurocognitive domains (aMCImd); (c) non-amnestic MCI single domain, defined by an impairment in one single domain other than memory (naMCIsd); and, finally, (d) a non-amnestic MCI multiple domains expressed by impairments in at least two neurocognitive domains, that do not include memory (naMCImd). These subtypes can lead to different epidemiology and pathogenesis (9). In particular, individuals affected by amnestic MCI are more likely to develop Alzheimer's disease [AD; (9)]. Besides memory, a commonly impaired domain in MCI (2, 10) and AD (11) is executive functioning.

Despite some guidelines being defined (7), there is great variability in the connotation of this diagnostic category. Clinical criteria include a cognitive impairment in one or more domains with a suggested cut-off equal to 1.0/1.5 SD below normative expectations (1). In the majority of cases, MCI diagnosis is advanced for patients with (i) memory problems corroborated by an informant, (ii) objective evidence of memory impairments, (iii) normal general cognitive functioning, (iv) intact functional abilities, and (v) no dementia (12). Among the different criteria for diagnosing MCI, the operationalization criteria proposed by Petersen et al. (7) have received strong support, thus becoming the most frequently used (12). However, various aspects remain unclear, and there are debates about them. For example, it is unclear whether cognitive decline is established only referred to age-specific norms or education-specific norms. Moreover, in contrast with Petersen's criteria (7), those proposed by Jak et al. (13) require that an individual obtains scores below 1 SD in at least two tests rather than one. These criteria are strongly associated with biomarkers for diagnosing AD, involving higher diagnostic stability and earlier identification of those who will turn into dementia (14).

MCI prevalence estimates are difficult to compare because of the differences among studies in the embraced definitions and the employed methodology (15). Moreover, there are numerous confounding variables, such as the sample's age and the number of schooling years, the applied diagnostic criteria, the assessed cognitive domains, and the assessment tools adopted for the diagnosis.

Many authors agree in considering MCI associated with a greater risk of dementia. Shah et al. (16) revealed that the rate of conversion to dementia in the MCI population is equal to 10–15% per year, while this rate amounts to 1–2% per year among the control group. Many authors reported a prevalence of MCI of 15–20% in the general population (17–19). However, prevalence estimates are variable and influenced by several factors: indeed, the presence or absence of MCI depends on the sensitivity and specificity of the tests used, population norms, and estimates of premorbid cognitive functioning (20). For this reason, prevalence estimates can range from 3 to 53.8% (21).

Establishing the prevalence of MCI is very important for clinical and research fields, as well as for the public health system. Indeed, the high numbers of people with dementia and cognitive disorders and their economic impact imply that an effective public health response should be a priority (20). Moreover, on a clinical level, it is important to identify the characteristics of MCI that allow us to predict the development of dementia. On the other hand, initial detection of cognitive decline can allow the early implementation of programs aimed at counteracting or slowing it down. To this end, it is important to have a clear picture of the prevalence of MCI and, consequently, of the diagnostic criteria used. Accordingly, this systematic review aims to analyze MCI prevalence and to explore the causes of its large heterogeneity further.

## Method

### Search strategy

This systematic review was conducted according to the PRISMA-Statement (22, 23). It has been performed by selecting articles published in international journals from January 1st, 1999, to January 20th, 2022, to include the year in which Petersen et al. (7) developed their MCI model. The databases utilized for the research were PsycInfo, PubMed, Scopus, and Web of Science. The review was based only on articles published in English and Italian. The search syntax can be found in Table 1.

**Table 1:** Search syntax.

**Database**	**Key words**	**Restrictions**	**N. of articles**	**Duplicates**	**Total**
PubMed	(((Mild Cognitive Impairment[Title/Abstract]) OR (MCI[Title/Abstract])) AND (Prevalence[Title/Abstract])) NOT ((Parkinson[Title/Abstract] OR Parkinson's disease[Title/Abstract] OR frontotemporal dementia[Title/Abstract] OR FTD[Title/Abstract] OR vascular dementia[Title/Abstract] OR VaD[Title/Abstract] OR Lewy body[Title/Abstract] OR Multiple Sclerosis[Title/Abstract]))	Publication years; 1999–2022; Languages: English, Italian; Species: Human; Age: Middle Aged (45–64 yrs), Aged (65+ yrs), 80 and over.	1,191		
Web of Science	(Mild Cognitive Impairment OR MCI) AND (prevalence) NOT (Parkinson OR Parkinson's disease OR frontotemporal dementia OR FTD OR vascular dementia OR VaD OR Lewy body OR Multiple Sclerosis)	Publication years: 1999–2022; Document type: Articles; Languages: English, Italian.	2,942		
Scopus	(Mild Cognitive Impairment OR MCI) AND (prevalence) AND NOT (Parkinson OR Parkinson's disease OR frontotemporal dementia OR FTD OR vascular dementia OR VaD OR Lewy body OR Multiple Sclerosis)	TITLE-ABS-KEY; DOCTYPE (ar); PUBYEAR > 1999; LANGUAGE “English” and “Italian”.	2,101		
PsycInfo	(Mild Cognitive Impairment OR MCI) AND (prevalence) NOT (Parkinson OR Parkinson's disease OR frontotemporal dementia OR FTD OR vascular dementia OR VaD OR Lewy body OR Multiple Sclerosis)	Publication years: 1999–2022; Reviewed by experts; Peer Reviewed Journal; English and Italian; Middle age (40–64 yrs), Aged (65 yrs and older), Very Old (85 yrs and older); Human; Journal Article; Exclude thesis.	461		
		**Total**	**6,695**	**3,574**	**3,121**

### Inclusion/exclusion criteria

According to the review aims, the following inclusion criteria were adopted: (I) controlled studies, cross-sectional and/or longitudinal, with the aim to evaluate MCI prevalence in the general population; (II) studies that specify and operationalize the diagnostic criteria and assessment tools for diagnosing MCI; (III) studies that precisely report the subject recruitment process.

The exclusion criteria were: (I) studies that included patients with head trauma, neurological disorders (e.g., epilepsy, multiple sclerosis, Parkinson's disease, etc.), metabolic diseases (e.g., diabetes or metabolic syndrome), autoimmune diseases (e.g., rheumatoid arthritis, Lupus, etc.), cardiovascular diseases (e.g., stroke or heart attack), oncological diseases, diagnosis of frontotemporal dementia, vascular dementia, dementia with Lewy bodies; (II) studies that included diagnoses of Cognitive Impairment Non Dementia-CIND, Age-Associated Memory Impairment-AAMI, Age-Associated Cognitive Decline-AACD; (III) studies that only adopted neurophysiological assessment tools, neuroimaging or biological markers for the diagnosis of MCI; (IV) studies that included only one gender in their sample (100% females or 100% males); (V) studies not containing the word “prevalence” in the title, in order to exclude articles that evaluated prevalence of MCI as a secondary goal, but that were not focused at evaluating MCI prevalence.

**Figure 1:**
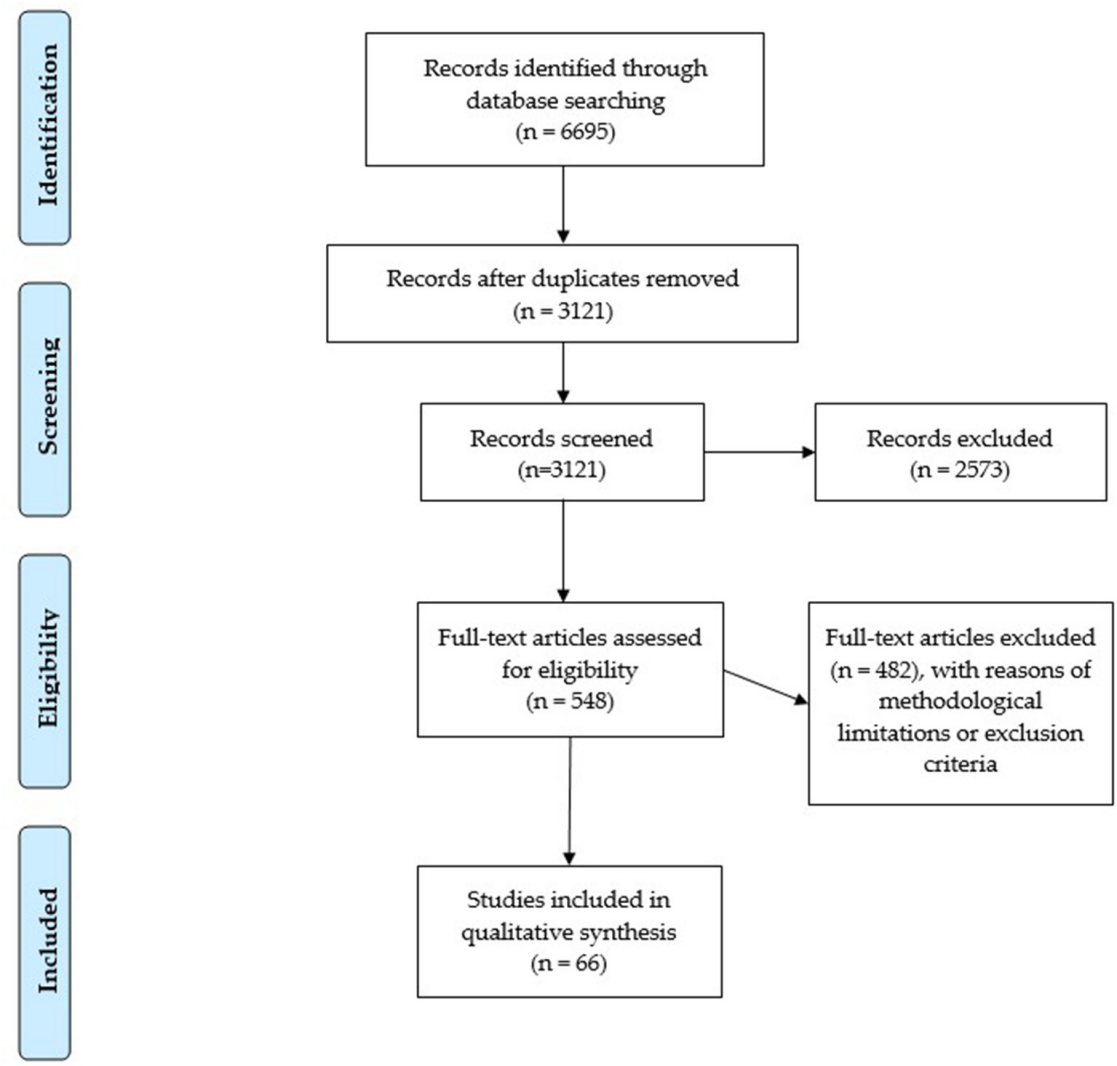
PRISMA flow diagram.

Two researchers independently screened the selected articles. The screening of the articles for titles and abstracts enabled the exclusion of those studies which were not relevant. This first screening resulted in 548 included articles. Subsequently, the full texts of the selected articles were read in order to evaluate their eligibility, thus resulting in 66 retained articles. The results are summarized in Figure 1.

### Data extraction and studies quality assessment

According to the PICOS method (22), the following information has been extracted from each of the included articles: authors and publication year; country; type of study; sample size, and participants' characteristics (age, schooling years, gender); adopted diagnostic criteria, employed assessment tools and cut-off values for MCI diagnoses; assessed cognitive domains; the prevalence of MCI.

Study quality (bias) was assessed following the guidelines developed by Loney et al. (24). This method consists of eight statements, and the scoring process implies assigning one point for each criterion achieved. Therefore, the maximum score is eight points. These statements are meant to evaluate sampling, response rate, condition measurements, sample size, the precision of estimates, study design, description of participants, setting, and non-responders (25).

### Publication bias

We attributed one point to each statement according to the assessment method developed by Loney et al. (24). From this process, we obtained 15 articles with eight points (maximum score), 34 with seven points, 13 studies were assigned 6 points, and only four articles received five points or less. These results are summarized in Table 2.

**Table 2:** Bias assessment.

**Points**	**Number of studies**
8	15
7	34
6	13
5	3
4	1
3	0
2	0
1	0

## Results

### Prevalence range

The sixty-six studies included in this systematic review involved 157,035 participants and reported a prevalence of MCI that ranges from 1.2% (26) up to 87% (27). This estimate drops to 0.6% (28) in the articles that evaluate only aMCI. This high heterogeneity is due to many reasons since these sixty-six articles vary in many aspects. These aspects are summarized in Table 3.

**Table 3:** Selected studies' characteristics and MCI prevalence.

**Author**	**Country**	**Study design**	**N**.	**Inclusion**	**Sex (% F)**	**Age**	**Schooling**	**Diagnostic criteria**	**Cut- off**	**Prevalence^a^**
Afgin et al. (29)	Israel	Longitudinal	944	≥65	50.6%	65–69: 39.4% 70–79: 46.4% ≥80: 14.1%	3 (±3)	Morris (30)	CDR = 0.5	32.1%
Alkhunizan et al. (31)	Saudi Arabia	Cross-sectional	171	≥60	43%	67 (±6)	Illiterate: 23.4% Non-illiterate: 76.6%	MoCA < 26 (32)	MoCA < 26	38.6%
Amer et al. (33)	Egypt	Cross-sectional	100	≥60	54%	Normal: 64.65 (±4) MCI: 67.75 (±4.99)	Illiterate: 16% Able to read and write: 14% Diploma: 42% Degree: 28%	MoCA < 26	MoCA < 26	32%
Anstey et al. (34)	Australia	Longitudinal	2,551	60–64 at wave 1	48.3%	70.60 (±1.50)	13.98 (±2.7)	Petersen (7)	(1) MMSE ≤ 25; (2) a score below the 5th percentile on immediate or delayed recall of the California Verbal Learning Test (immediate or delayed score of < 4and < 2, respectively); (3) a score below the 5th percentile on either of the following two tests: Symbol-Digit Modalities Test (<33) or Purdue Pegboard with both hands (<8) or reaction time (third set of 20 trials) (>0.31 s).	7.68%
Busse et al. (35)	Germany	Longitudinal	929	≥75	75.9%	81.5 (±4.9)	Low: 22.4% Medium: 63.6% High: 13.6% Illiterate: 0.4%	aMCI: Petersen (7) aMCI-modified: no criteria regarding subjective cognitive complaints	1 SD	aMCI: 3.1%; aMCI– modified: 5.1%.
Busse et al. (36)	Germany	Longitudinal	929	≥75	75.9%	81.5 (±4.9)	Low: 22.4% Medium: 63.6% High: 13.6% Illiterate: 0.4%	aMCI: Petersen (7) aMCI-modified: no criterion regarding subjective cognitive complaints	1–1.5–2 SD	aMCI: 1 DS: 15% 1.5 DS: 8.5% 2 DS: 5% aMCI-modified: 1 DS: 32.5% 1.5 DS 16.8% 2 DS 9.3%
Chuang et al. (37)	Taiwan	Cross-sectional	470	≥75	61.3%	71.2 (±5.4)	7.9 (±4.4)	Albert (1)	1.5 SD	17.3%
Clark et al. (38)	United States	Longitudinal	476	-	70%	53.8 (±6.7)	16.2 (±2.9)	Jak (13) Criterion 1: At least one test blows the cut-off Criterion 2: At least two tests below the cut-off for one cognitive domain Cut-off values follow both standard and robust norms	1.5 SD	Criterion 1 – standard norms: 18% Criterion 1 – robust norms: 49% Criterion 2 – standard norms: 3% Criterion 2 – robust norms: 20%
Dimitrov et al. (39)	Bulgaria	Cross-sectional	540	≥65	58.7%	73 (±5.5)	–	Petersen's criteria modified by Portet (40)	MMSE < 25	6.7%
Ding et al. (41)	China	Cross-sectional	3,141	≥60	54.2%	72.3 (±8.1)	11.6 (±4.4)	Petersen (3)	1.5 SD	20.1%
Dlugaj et al. (42)	Germany	Cross-sectional	656	50–80	47%	68.9 (±6.6)	≤ 10: 18.6% 11–13: 61.4% ≥14: 20%	MCI- original criteria (2.90). MCI- modified criteria: no criterion regarding subjective cognitive complaints	1 SD	MCI – original: 7.8% MCI – modified: 12.1%
Fernández - Blázquez et al. (43)	Spain	Longitudinal	1,180	≥70	63.6%	74.9 (±3.9)	Illiterate: 20.4% Primary education: 30.6% Secondary education: 23.7% Higher education: 25.4%	Albert (1)	CDR = 0.5	7%
Ganguli et al. (44)	United States	Cross-sectional	1,982	≥65	61.1%	77.6 (±7.4)	Less than high school: 13.8% High school: 45.1% More than high school: 41.1%	Criterion 1: CDR = 0.5 Criterion 2: *Ad hoc* Criterion 3: Petersen (3) -aMCI Criterion 4: Winblad (45)	1 SD	Criterion 1: 27.6% Criterion 2: 34.7% Criterion 3: 2.27% Criterion 4: 17.61%
Gavrila et al. (46)	Spain	Cross-sectional	1,074	≥65	55.7%	73.9 (±6.8)	Illiterate: 7.8% Able to read and write: 19.7% Primary: 36.3% Secondary: 25.5% >Secondary: 10.7%	Caracciolo (47)	1.5 SD	aMCI: 8.7%
GjØra et al. (48)	Norway	Cross-sectional	9,663	≥70	54.4%	77.9 (±6.4)	< 10: 41.8% 11–12: 34% >12: 24.2%	DSM-5	-	35.3%
González et al. (49)	United States	Longitudinal	6,377	≥45	55%	63 (±8)	>12: 40%	Albert (1)	1 SD	9.8%
Han et al. (50)	South Korea	Cross-sectional	755	≥65	–	–	–	Petersen (3)	1.5 SD	31.85%
Hanninen et al. (51)	Finland	Cross-sectional	806	60–76	60.2%	68.1 (±4.5)	9.1 (±3.4)	Petersen (7)	1.5 SD	5.3%
Jia et al. (17)	China	Cross-sectional	1,0276	≥65	Urban: 56.8% Rural: 58.2%	Urban: 65–69: 29% 70–74: 34.3% 75–79: 22.9% ≥80: 13.8% Rural: 65–69: 34.7% 70–74: 27.3% 75–79: 22.7% ≥80: 15.3%	Urban: < 1: 17.7% 1–6: 32.7% 7–9: 19% ≥10: 29.9% Rural: < 1: 48.2% 1–6: 38.8% 7–9: 9.8% ≥10: 3.1%	Petersen (3) e Winblad (45)	1.5 SD	20.8%
Juncos-Rabadan et al. (52)	Spain	Cross-sectional	580	≥50	69.1%	50–54: 8.6% 55–59: 9.5% 60–64: 16.2% 65–69: 16.6% 70–74: 18.6% 75–79: 17.2% 80–84: 8.8% 85–90: 4.5%	0–4: 15.5% 5–8: 46.4% 9–12: 19.7% 13+: 18.4%	Petersen (3)	1.5 SD	29.1%
Katz et al. (53)	United States	Longitudinal	1,818	≥70	–	–	–	Artero (54)	1.5 SD	21.5%
Khedr et al. (55)	Egypt	Cross-sectional	691	≥60	–	–	–	–	1 SD	aMCI: 1.74%
Kim et al. (56)	South Korea	Cross-sectional	1,673	≥65	60.2%	–	–	Petersen (3)	1.5 SD	24.1%
Kochan et al. (57)	Australia	Cross-sectional	987	70–90	English speaking background: 56% Non-English speaking background: 50.6%	English speaking background: 78.55 (±4.75) Non-English speaking background: 79.85 (±4.90)	English speaking background: 11.64 (±3.51) Non-English speaking background: 11.52 (±3.29)	Petersen (3)	1–1.5–2 SD	4–70%
Kumar et al. (58)	Australia	Cross-sectional	2,551	60–64	48.3%	70.60 (±1.50)	13.98 (±(2.7)	Petersen (7)	(1) MMSE ≤ 25; (2) a score below the 5th percentile on immediate or delayed recall of the California Verbal Learning Test (immediate or delayed score of < 4 and < 2, respectively); (3) a score below the 5th percentile on either of the following two tests: Symbol-Digit Modalities Test (<33) or Purdue Pegboard with both hands (<8) or reaction time (third set of 20 trials) (>0.31 s).	3.7%
Lara et al. (59)	Spain	Cross-sectional	3,625	≥50	53.6%	66.26	Illiterate: 32.3% Primary education: 31.3% Secondary education: 25.6% Higher education: 10.8%	Albert (1)	1 SD	9.6%
Lee et al. (60)	South Korea	Cross-sectional	714	≥65	57.8%	71.9 (±5.7)	≤ 6: 50.7% ≥7: 49.3%	Petersen (3)	1.5 SD	27.6%
Li et al. (61)	China	Cross-sectional	1,020	≥55	63.3%	63.9 (±6.6)	11.6 (±2.9)	Petersen (3)	1.5 SD	15.7%
Limongi et al. (62)	Italy	Longitudinal	2,337	≥65	58.3%	74	5	Petersen's modified criteria (42)	1 SD	21.6%
Lindgren et al. (63)	Finland	Cross-sectional	1,772	75–81	49.7%	Women: 73.7 (±1.4) Men: 73.9 (±1.6)	Women: 8.6 (±3.1) Men: 8.5 (±3.2)	Knopman (64)	Score 28–31	9.3%
Liu et al. (65)	Taiwan	Cross-sectional	10.432	≥65	52%	Urban: 76.5 (±7.3) Suburban: 76.2 (±6.8) Rural: 76.1 (±6.3)	Urban: 0: 17.9% ≤ 6: 43.1% 7–12: 25.9% >12: 13.1% Suburban: 0: 30.6% ≤ 6: 43.9% 7–12: 17.9% >12: 7.6% Rural: 0: 42.6% ≤ 6: 47.8% 7–12: 7.8% >12: 1.9%	–	–	Urban: 15.11% Suburban: 16.67% Rural: 20.29%
Liu et al. (66)	Singapore	Cross-sectional	973	≥65	72.87%	MCI: 69.39 (±6.67) Normal: 67.65 (±5.52)	MCI: 0: 52.5% 1–6: 26.2% >6: 21.3% Normal: 0: 35.4% 1–6: 33.8% >6: 30.8%	Albert (1)	1.5 SD	12.5%
Lopez et al. (67)	United States	Longitudinal	CHS Cohort: 2,470 Pittsburgh Cohort: 599	-	CHS Cohort: 60.9% Pittsburgh Cohort: 59.6%	CHS Cohort: < 75: 15.7% 75–79: 49.4% 80–84: 23.6% 85+: 11.2% Pittsburgh Cohort: < 75: 14.5% 75–79: 51.4% 80–84: 25% 85+: 9%	CHS Cohort: < high school: 51.6% >high school: 48.3% Pittsburgh Cohort: < high school: 43.9% >high school: 56.1%	–	1.5 SD	CHS Cohort: 18.8% Pittsburgh Cohort: 21.7%
Lopez-Anton et al. (68)	Spain	Cross-sectional	4,803	≥55	Non cases: 43.93% P-MCI: 61.60% DSM-MCI: 69.48%	Non cases: 71.54 (±8.97) P-MCI: 73.54 (±8.69) DSM-MCI: 80.53 (±8.75)	Non cases: 7.81 (±3.97) P-MCI: 6.49 (±3.29) DSM-MCI: 6.32 (±3.18)	Petersen (7) / DSM-5	-	aMCI – Petersen: 7.05% MCI DSM-5: 3.36%.
Luck et al. (69)	Germany	Longitudinal	3,242	≥75	65.6%	80.1 (±3.6)	Low: 62% Middle: 27.3% High: 10.6%	Original criteria (45) Modified criteria: no criterion regarding subjective cognitive complaints	1 SD	Original criteria: 5.4% Modified criteria: 25.2%
Ma et al. (70)	China	Cross-sectional	5,214	≥65	56.1%	72.13 (±4.22)	6.34 (±7.26)	Petersen (3)	1.5 SD	11.33%
Meguro et al. (71)	Japan	Cross-sectional	1,501	≥65	–	CDR 0: 72.7	CDR 0: 8.4	Petersen (7)	1.5 SD	4.9%
						CDR 0.5: 74.8	CDR 0.5: 7.6			
						CDR >1: 78.7	CDR >1: 7.8			
Mohan et al. (72)	India	Cross-sectional	426	≥60	62%	69.9 (±7.9)	6.78 (±4)	Portet (40)	1 SD	26.06%
Mooi and Hamid (73)	Malaysia	Cross-sectional	2,112	≥60	51.4%	68.8 (±6.1)	Illiterate: 19% Primary education: 50.2% Secondary education: 17.3% Higher education: 5.2%	Shahar (74)	MMSE ≤ 21	68%
Moretti et al. (75)	Italy	Cross-sectional	7,930	≥60	60.3%	72.6 (±8.2)	0–3:45.6% 4–8: 43.3% >8: 11.1%	Winblad (45) Definition A: no criterion regarding subjective cognitive complaints Definition B: original criteria	1.5 SD	MCI-A: 24.5% MCI- B: 6%
Noguchi-Shinohara, et al. (76)	Japan	Cross-sectional	650	≥65	59.5%	76.4 (±7.3)	9.6 (±2.3)	Winblad (45)	CDR = 0.5	16.4%
Ogunniyi, et al. (77)	Nigeria	Cross-sectional	642	≥65	Normal: 68.3% MCI: 80.2% Dementia: 41.2%	–	–	Petersen (9)	–	18.4%
Paddick et al. (78)	Tanzania	Cross-sectional	296	≥70	–	–	–	Winblad (45)	–	6.3%
Parlevliet et al. (79)	Netherlands	Cross-sectional	2,254	≥55	55.6%	65 (±7.5)	–	Krabbe (80)	1 SD	Turkish: 3% Moroccan - Arabic: 10.1% Moroccan – Berber: 9.4% Surinamese - Hindustani: 11.9% Surinamese – Creole: 5.9% Native Dutch: 3.3%
Peltz et al. (81)	United States	Cross-sectional	420	≥90	66%	93.2	High school or less: 23.1% < College graduate: 32.4% College graduate or more: 44.5%	Petersen (82)	1.5 SD	16.6%
Petersen et al. (83)	United States	Longitudinal	1,969	70–89	49.1%	70–79: 53.8% 80–89: 46.2%	< 9: 7.2% 9–12: 39.5% 13–16: 36.7% >16: 16.7%	Petersen (3)	1 SD	16%
Radford et al. (84)	Australia	Cross-sectional	336	≥60	59.5%	66.6 (±6.3)	< 10: 61% 10 or more: 39%	Winblad (45)	MMSE ≤ 26, mKICA ≤ 35, and/or RUDAS ≤ 25	17.7%
Rao et al. (85)	China	Cross-sectional	2,111	≥65	59.5%	Men: 75.7 (±6.5)	Rural: 2.16 ± 2.99	Petersen (3)	1.5 SD	14.2%
						Women: 76.1 (±6.8)	Urban: 4.77 ± 4.79			
Rentería et al. (86)	Mexico	Cross-sectional	1,807	≥55	58.9%	67.2 (±8.4)	5.7 (±4.5)	Jak (13)	1.5 SD	34.4%
Robertson et al. (27)	United States	Longitudinal	1,721	≥65	59.5%	78.2 (±7.3)	15.7 (±3.0)	Trittschuh (87)	1–1.5 SD	43–87%
Ruan et al. (88)	China	Cross-sectional	5,175	≥60	53.4%	60–69: 45.8%	< 6: 9.4%	–	Scores 6–7	9.67%
						70–79: 37.4%	6–12: 66.8%			
						≥80: 16.8%	>15: 15.9%			
Sasaki et al. (89)	Japan	Cross-sectional	1,443	≥65	58.9%	73.6 (±5.8)	10 (±2.6)	–	1 – 1.5 SD	1 DS: 38.9% 1.5 DS: 18.9%
Shahnawaz et al. (90)	Australia	Cross-sectional	767	70–90	56.5%	78.53 (±4.68)	11.61 (±3.51)	Winblad (45)	1.5 SD	38.9%
Shimada et al. (19)	Japan	Cross-sectional	5,025	≥65	51%	–	–	Jungwirth (91)	1.5 SD	18.8%
Sosa et al. (28)	Cuba, Dominican Republic, Peru, Mexico, Venezuela, Puerto Rico, China, and India	Cross-sectional	15,376	≥65	Cuba: 64.4% Dominican Republic: 65.3% Peru: 60.7% Mexico: 62.8% Venezuela: 63% Puerto Rico: 67% China: 56% India: 54%	Cuba; 65–69: 28.2% 70–74: 28.2% 75–79: 22.2% 80+: 21.2% Dominican Republic: 65–69: 28.9% 70–74: 27.3% 75–79: 19.5% 80+: 24.2% Peru: 65–69: 30.5% 70–74: 26.9% 75–79: 20.8% 80+: 21.8% Mexico: 65–69: 29.5% 70–74: 30.3% 75–79: 21.1% 80+: 19.1% Venezuela: 65–69: 44.7% 70–74: 24.7% 75–79: 17.6% 80+: 13% Puerto Rico: 65–69: 22.6%	Cuba: Illiterate: 2.1% Some education: 20.9% Primary education: 33% Secondary education: 26% Higher education: 17.9% Dominican Republic: Illiterate: 17.8% Some education: 51.8% Primary education: 19.1% Secondary education: 7.1% Higher education: 3.7% Peru: Illiterate: 5.8% Some education: 12% Primary education: 37%	Petersen (9)	1.5 SD	aMCI Cuba: 1.5% Dominican Republic: 1.3% Peru: 2.6% Mexico: 2.8% Venezuela: 1% Puerto Rico: 3% China: 0.6% India: 4.6%
						70–74: 24.9% 75–79: 24.7% 80+: 27.9% China: 65–69: 33.9% 70–74: 31.5% 75–79: 20.7% 80+: 13.9% India: 65–69: 39% 70–74: 33.5% 75–79: 16.1% 80+: 11.2%	Secondary education: 27.5% Higher education: 17% Mexico: Illiterate: 25.2% Some education: 44% Primary education: 18.5% Secondary education: 6.4%			
							Secondary education: 6.4% Higher education: 5.7% Venezuela: Illiterate: 7.3% Some education: 22.4% Primary education: 50.2% Secondary education: 14.4% Higher education: 5.1% Puerto Rico: Illiterate: 2.7% Some education: 17.7% Primary education: 20.2%			
							Secondary education: 37.5% Higher education: 21.7% China: No education: 36.9% Some education: 12.2% Primary education: 26.4% Secondary education: 17.8% Higher education: 6.7% India: Illiterate: 51.9% Some education: 22.8% Primary education: 16.7% Secondary education: 6.1% Higher education: 2.4%			
Su et al. (92)	China	Cross-sectional	796	≥60	62.7%	71.4 (±6.8)	0: 13.7% 1–6: 23.9% 7–9: 31.3% 10–12: 21% ≥13: 10.2%	Petersen (3)	MMSE ≤ 17 for illiterates; MMSE ≤ 20 for primary school graduates (≥6 years of education), MMSE ≤ 24 for junior school graduates or above (≥9 years of education)	18.2%
Teh et al. (26)	Singapore	Cross-sectional	2,165	≥60	-	-	-	Winblad (45)	1.5 SD	1.2%
Tognoni et al. (93)	Italy	Cross-sectional	1,600	≥65	59.6%	74.65 (±7.26)	Illiterate: 1.8% At least 3 years: 14% University degree: 0.7%	Petersen (7)	MMSE ≤ 24 CDR = 0.5	aMCI: 4.9%
Tsolaki et al. (94)	Greece	Cross-sectional	443	≥65	In line with diagnoses made with method 4: Normal group: 32.2% MCI group: 35.3% Depression group: 62.7% MCI with depression group: 73.7% Dementia group: 55.6% Dementia with depression group: 68.8%	In line with diagnoses made with method 4: Normal group: 73 (6.8) MCI group: 75.7 (±7.1) Depression group: 76.1 (±6.4) MCI with depression group: 77.8 (±7) Dementia group: 75.7 (±6.9) Dementia with depression group: 78.4 (±7.7)	In line with diagnoses made with method 4: Normal group: 4.1 (±2.9) MCI group: 2.7 (±2.4) Depression group: 3.8 (±2.9) MCI with depression group: 3.9 (±2.7) Dementia group: 5 (±3.5) Dementia with depression group: 3.2 (±2)	Method 1: *Ad hoc* Method 2: Same criteria as method 1 but MMSE score is adjusted for age and schooling Method 3: Petersen (95) Method 4: Same criteria as method 1 but MMSE score is adjusted for age and schooling	MMSE/HINDE < 28 and >24	Method 1: MCI: 16.5 % MCI with depression: 12.9 % Method 2: MCI: 5.9 % MCI with depression: 6.3 % Method 3: MCI: 20.1 % MCI with depression: 21.9 % Method 4: MCI: 15.3 % MCI with depression: 8.6 %
Tsoy et al. (96)	Kazakhstan	Cross-sectional	662	≥60	75.7%	70	< high school: 1.96% High school: 10.42% College: 32.33% University: 55.29%	Winblad (45)	MoCA ≤ 26	30%
Vlachos et al. (97)	Greece	Longitudinal	1,960	≥65	59.4%	73.46 (±5.471)	7.95 (±4.940)	Petersen (3)	1.5 SD	13.11%
Welstead et al. (98)	United Kingdom	Longitudinal	567	Wave 3: 76 years	MCI: 38%	MCI: 76.21 (±0.66)	MCI: 10.76 (±1.16)	Albert (1)	1.5 SD	15%
					Normal: 48%	Normal: 76.25 (±0.68)	Normal: 10.81 (±1.13)			
Wu et al. (99)	China	Cross-sectional	2,065	≥60	59.4%	76.25 (±6.6)	>7: 72.1%	–	MMSE score < 17 for illiterate subjects, < 20 for subjects with 1–6 years of education and < 24 for subjects with < 7 years of education.	15.4%
Xu et al. (100)	China	Cross-sectional	2,426	≥60	60.7%	69.1 (±6.8)	0–6: 43.2% 7–12: 44.8% >12: 12%	Petersen (7)	1.5 SD	21.3%
Yang et al. (101)	China	Cross-sectional	925	≥65	54%	71.16 (±4.41)	Dementia: ≤ 6: 83.5% 7–9: 13.6% 10–12: 1.9% >12: 1% MCI: ≤ 6: 65.9% 7–9: 31.2% 10–12: 2.2% >12: 0.7% Normal: ≤ 6: 41.6% 7–9: 48.9% 10–12: 5.7% >12: 3.8%	Petersen (3)	1.5 SD	29.8%

a If available, standardized prevalence rates have been reported.

### Type of studies

Most of these studies are cross-sectional, except for 18 articles out of 66 that are longitudinal. Moreover, 57.6% of them have a sample size larger than 1,000 participants. In the risk of bias assessment, according to the guidelines (24), we considered a sample of fewer than 300 participants affected by bias. However, only three studies have included < 300 subjects (31, 33, 78).

### Age

**Figure 2:**
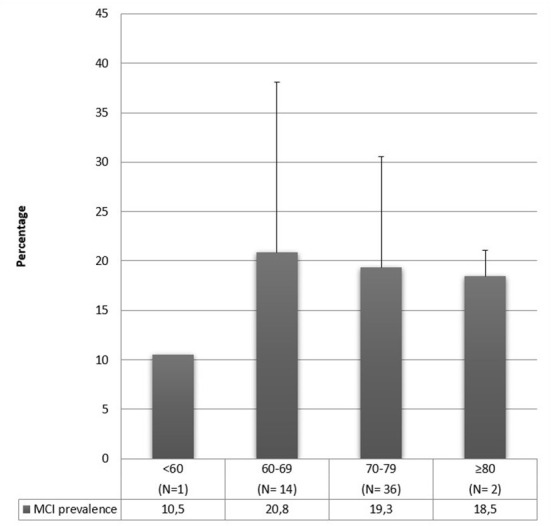
MCI mean prevalence rates according to age.

The majority of these studies included participants over 60 years old. The authors that include younger samples [≥45, ≥50 or ≥55; (42, 49, 52, 59, 61, 68, 79, 86)] found a prevalence range that goes from 3 to 34.4%. This range is lower than the whole range, implying that a lower prevalence could be caused by sampling younger people since cognitive deficits increase with aging. However, even among authors who included young adults in their sample, it is easily noticeable that there is great variability in MCI prevalence estimates (from 3 to 34.4%). This aspect indicates that age is not the only variable that affects the prevalence results. Moreover, as can be observed in Figure 2, people aged 60–69 show a slightly higher prevalence of MCI when compared with older people. This aspect contradicts what was stated above, but many factors can be explained it. First, this prevalence estimate by age group was made considering the average age of the participants; however, the age range is generally not reported, nor is the age composition of the sample reported (e.g., if the mean age of the sample is 65 years old, it is not clear how many people aged 60 and how many of 85 contribute to determining this mean). Second, the outcome may be due to the extreme variability in diagnostic criteria and tests used to assess cognitive decline. Third, studies generally do not report whether other factors may influence cognitive decline (e.g., depression, anxiety, poor sleep quality) and whether the outcome may depend on medications (e.g., antidepressants, hypnotics, rivastigmine), which are likely to be hired more at a later age. Fourth, older people could already be progressed to dementia; therefore, MCI prevalence rates are lower when age increases. Finally, Figure 2 includes 53 out of 66 studies because 6 were excluded because of an aMCI diagnosis, and 7 studies were excluded since they did not report the mean age of the participants. However, it is not possible to draw definite conclusions.

### Gender

Gender composition varies among studies, with a female share ranging from 43 to 75.9%. Some of our studies included a vast majority of women [≥70%; (35, 36, 38, 66, 96)] and found a prevalence range that goes from 3 to 49%. This range is lower than the total range, which could mean that women present lower MCI prevalence rates than men, according to Petersen's findings (83). However, most studies did not report the prevalence separately for women and men, so it is impossible to draw definitive conclusions.

### Schooling years

**Figure 3:**
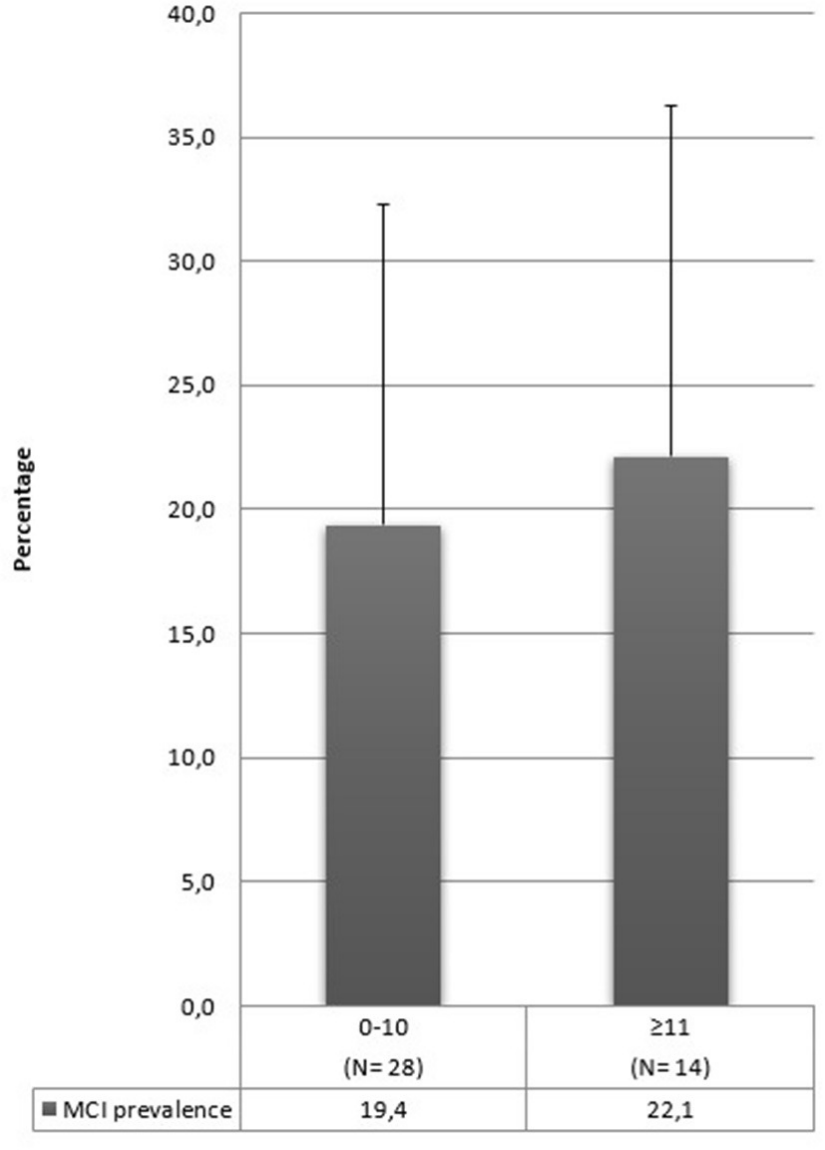
MCI mean prevalence rates according to schooling years.

In our review, many studies included illiterate people in their sample (17, 28, 31, 33, 35, 36, 43, 46, 59, 65, 66, 73, 92). The prevalence range found by these studies goes from 6 to 68%, but these rates drop to 0.6% (28) in the articles that evaluate only aMCI. It is conceivable that MCI prevalence can increase by including illiterate people in the sample, but it is impossible to corroborate this hypothesis with the results of this review. Moreover, some studies have not specified the schooling years of their sample, and this aspect complicates the interpretation of our results further. In Figure 3, we synthesized schooling years into two categories: 10 or fewer years or above 10. Prevalence rates are similar in these two categories, and this information confirms that it is difficult to establish whether this aspect contributes to the development of MCI.

### Geographical region

**Figure 4:**
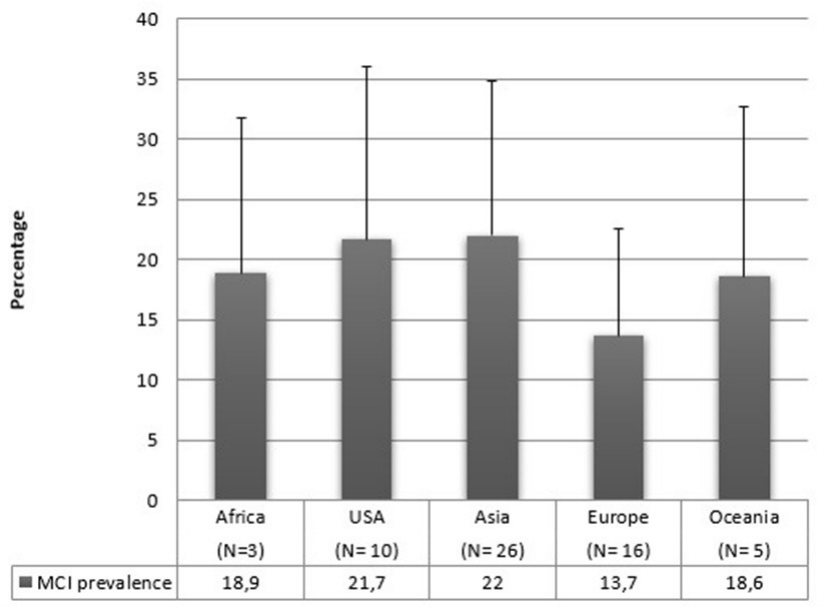
MCI mean prevalence rates according to the geographical region of samples.

Regarding geographical distribution, 26 studies were conducted in Asia, 20 in Europe, 10 in America, 5 in Oceania, 4 in Africa, and one study included people from both America and Asia. Figure 4 reports the mean prevalence of MCI by geographic distribution. It can be seen that the standard deviations are high, and the majority of articles are from Asia or Europe, and we do not have sufficient data on the other areas. Therefore, it is impossible to draw conclusions on this matter in our review.

### MCI criteria and operationalizations

There are problems linked to differences in the methodologies of the studies. Many different criteria have been used to diagnose MCI. Furthermore, researchers that utilize the same criteria often operationalize them differently and use diverse neuropsychological tasks.

Various tasks have been used to evaluate cognitive decline. Some of them measured global cognitive functioning, such as CAMCOG (Cambridge Cognitive Examination), CDR (Clinical Dementia Rating Scale), CERAD (Consortium to Establish a Registry for Alzheimer's Disease), CSI-D (Community Screening Instrument for Dementia), MMSE (Mini-Mental State Examination), MoCA (Montreal Cognitive Assessment), WAIS (Wechsler Adult Intelligence Scale), SIDAM (A Structured Interview for the diagnosis of dementia of the Alzheimer type, Multi-infarct dementia and dementias of other etiology according to ICD-10 and DSM-III-R); others measure different cognitive domains (such as intelligence, memory, attention, executive functioning, language, praxia, visuospatial abilities, and processing speed). All the neuropsychological tasks used in the selected articles are reported in Table 4.

**Table 4:** Assessed cognitive domains and neuropsychological tests^a^.

**Author**	**Diagnostic criteria**	**Assessed cognitive domains**								
		**Global functioning**	**Intelligence**	**Memory**	**Attention**	**Executive functions**	**Language**	**Praxia**	**Visuospatial ability**	**Processing speed**
Afgin et al. (29)	CDR= 0.5 (30)	CDR	–	–	–	–	–	–	–	–
Alkhunizan et al. (31)	MoCA < 26 88	MoCA	–	–	–	–	–	–	–	–
Amer et al. (33)	MoCA < 26	MMSE MoCA	–	–	–	–	–	–	–	–
Anstey et al. (34)	Petersen (7)	MMSE SDMT CDR	–	RAVLT CVLT	–	TMT A-B VF CDT	BNT Short Form WAB	CERAD	–	–
Busse et al (a) (35)	aMCI: Petersen (7) aMCI-modified: no criterion regarding subjective cognitive complaints	SIDAM CDR	–	–	–	–	–	–	–	–
Busse et al. (b) (36)	aMCI: Petersen (7) aMCI-modified: no criterion regarding subjective cognitive complaints	SIDAM CDR	–	–	–	–	–	–	–	–
Chuang et al. (37)	Albert (1)	MMSE CDR		WMS LM	DST DSST WAISIII Symbol Searching	Color Trails Test Stroop Color Word Test Semantic VF				
Clark et al. (38)	Jak (13) Criterion 1: At least one test blows the cut-off Criterion 2: At least two tests below the cut-off for one cognitive domain	–	–	WMS-R LM RAVLT BVMT-R	–	WAIS-R DSST Stroop Color- Word Interference TMT Part B	–	–	–	–
Dimitrov et al. (39)	Petersen's criteria modified by Portet (40)	MMSE CERAD	–	MIS MIS (alternative form)	–	–	–	–	–	–
Ding et al. (41)	Petersen (3)	CDR somministrato ad un proxy MMSE	–	Stick Test AVLT Modified FOME Renminbi Test	TMT A&B Renminbi Test	Conflicting Instructions Task (Go/No-Go Task) Modified Common Objects Sorting Test TMT A&B	Modified Common Objects Sorting Test Renminbi Test	–	Stick Test	–
Dlugaj et al. (42)	MCI- original criteria (2.90). MCI- modified criteria: no criterion regarding subjective cognitive complaints	ADAS	–	–	–	VF [2 subtests with a formal lexical category ('S' and 'G-R') and 2 subtests with a semantic category ('food' and 'clothes-flowers')]	–	–	–	NCT of the Nürnberg Gerontopsychological Inventory
Fernández -Blázquez et al. (43)	Albert (1)	CDR	–	–	–	–	–	–	–	–
Ganguli et al. (44)	Criterion 1: DR=0.5 Criterion 2: *Ad hoc* Criterion 3: Petersen (3) -aMCI Criterion 4: Winblad (45)	MMSE		WMS-R LM WMS-R VR FOME with Semantic Interference	TMT A DSF	TMT B CDT VF (letter P&F)	BNT VF Categories (animals) IU Token Test	–	WAIS-III BD	TMT A DSF
Gavrila et al. (46)	Caracciolo (47)	MMSE CAMDEX Blessed Dementia Scale Reisberg GDS	–	–	–	–	–	–	–	–
GjØra et al. (48)	DSM-5	MoCA CDR IADL	–	CERAD WLM	–	–	–	–	–	–
González et al. (49)	Albert (1)	SIS NIHTB PVT eCog12	–	B-SEVLT	–	WF DSST TMT, parts A&B	–	–	–	WF DSST
Han et al. (50)	Petersen (3)	MMSE CERAD-K-C CERAD-K-N	–	–	–	DST FAB	–	–	–	–
Hanninen et al. (51)	Petersen (7)	CDR MMSE	–	BSRT WMS VR WMS-R LM	VF TMT, parts A&B	VF TMT, parts A&B	Abbreviated (15 items) BNT	–	–	–
Jia et al. (17)	Petersen (3) Winblad (45)	MMSE MoCA CDR	–	WHO-UCLA AVLT	–	TMT part b.	VF (category, animals)	–	CDT	–
Juarez-Cedillo et al. (102)	Petersen (95)	MMSE ADAS CERAD CDR	–	MIS RBMT SKT SBT	SKT SBT	–	Semantic VF	–	CDT	–
Juncos -Rabadan et al. (52)	Petersen (3)	MMSE CAMCOG-R	–	CVLT	–	–	–	–	–	–
Katz et al. (53)	Artero (54)	BIMC CDR	–	FCSRT WMS-R LM	TMT part A DST	TMT part B FAS	Category VF (animals, vegetables, fruits) BNT	–	WAIS-III BD DSST	–
Khedr et al. (55)	–	MMSE CDR	–	MES WMS-III LM	–	MES	–	–	–	–
Kim et al. (56)	Petersen (3)	MMSE-KC CERAD-K-C CERAD-K-N	–	–	–	–	–	–	–	–
Kochan et al. (57)	Petersen (3)	–	NART	WMS-III LM RAVLT BVRT	TMT part A DSST	TMT part B FAS	BNT 30 items semantic VF (Animals)	–	WAIS-III BD	TMT part A DSST
Kumar et al. (58)	Petersen (7)	MMSE SDMT CDR	–	RAVLT CVLT	–	TMT A-B VF CDT	BNT Short Form WAB	CERAD	–	–
Lara et al. (59)	Albert (1)	–	–	CERAD	DST	–	CERAD	–	–	–
Lee et al. (60)	Petersen (3)	CERAD-K-C CERAD-K-N	–	–	DST	-	Lexical VF	–	–	–
Li et al. (61)	Petersen (3)	–	–	AVLT ROCF	TMT part A SDMT	TMT part B Stroop test	Category VF BNT	–	ROCF copy test CDT	–
Limongi et al. (62)	Dlugaj (42)	MMSE CDT ADAS-Cog	–	ROCF famous faces naming test SRT	–	TMT	Phonemic VF semantic VF	–	–	–
Lindgren et al. (63)	Knopman (64)	TICS-m	–	–	–	–	–	–	–	–
Liu et al. (65)	Albert (1)	MMSE CDR ADL IADL	–	–	–	–	–	–	–	–
Liu et al. (66)	Albert (1)	MMSE MoCA CDR GDS GAI	–	RAVLT	SDMT DST	Color Trails	semantic VF	–	WAIS-III BD	–
Lopez et al. (67)	–	3MS TICS IQCODE	–	BVRT	DSST	–	–	–	–	–
Lopez-Anton et al. (68)	Petersen (7) / DSM-5	MMSE	–	–	–	–	–	–	–	–
Luck et al. (69)	Original criteria (45) Modified criteria: no criterion regarding subjective cognitive complaints	SIDAM	–	–	–	–	–	–	–	–
Ma et al. (70)	Petersen (3)	WMS-R WAIS-R MMSE	–	–	–	–	–	–	–	–
Meguro et al. (71)	Petersen (7)	CASI MMSE	–	–	–	–	–	–	–	–
Mohan et al. (72)	Portet (40)	ACE	–	–	–	–	–	–	–	–
Mooi and Hamid (73)	Shahar (74)	MMSE	–	–	–	–	–	–	–	–
Moretti et al. (75)	Winblad (45) Definition A: no criterion regarding subjective cognitive complaints Definition B: original criteria	MMSE GDS semi-structured neurologic checklist	–	–	–	–	–	–	–	–
Noguchi-Shinohara, et al. (76)	Winblad (45)	MMSE CDR	–	–	–	–	–	–	–	–
Ogunniyi, et al. (77)	Petersen (9)	IDEA	–	–	–	–	–	–	–	–
Paddick et al. (78)	Winblad (45)	CERAD	–	–	–	–	–	–	–	–
Parlevliet et al. (79)	Krabbe (80)	CCD	–	–	–	–	–	–	–	–
Peltz et al. (81)	Petersen (82)	MMSE	–	CVLT	–	DSB	Category VF	CERAD Constructions	–	–
Petersen et al. (83)	Petersen (3)	CDR all'informatore STMS	–	WMS-R LM WMS-R VR AVLT delayed percent retention	–	TMT part B DSST	BNT category VF		Picture completion WAIS-R BD	–
Radford et al. (84)	Winblad (45)	MMSE	–	–	–	–	–	–	–	–
Rao et al. (85)	Petersen (3)	MoCA MMSE CDR	–	WHO-UCLA AVLT	–	TMT part B	Semantic VF (category, animals)	–	CDT	–
Shimada et al. (19)	Petersen (9)	MMSE NCGGFAT	–	–	–	–	–	–	–	–
Sosa et al. (28)	Petersen (9)	CSI “D”	–	CERAD ten-wordlist learning task	–	–	–	–	–	–
Su et al. (92)	Petersen (3)	MMSE CDR	–	–	–	–	–	–	–	–
Teh et al. (26)		CSI'D	–	CERAD WLM CERAD WLR	–	TMT, parts A&B figure copy	BNT VF	–	–	–
Tognoni et al. (93)	Petersen (7)	MMSE CAMDEX CDR MDB	–	–	–	–	–	–	–	–
Tsolaki et al. (94)	Method 1: *Ad hoc* Method 2: Same criteria as method 1 but MMSE score is adjusted for age and schooling Method 3: Petersen (95) Method 4: Same criteria as method 1 but MMSE score is adjusted for age and schooling	MMSE o HINDI for illiterate	–	–	–	–	–	–	–	–
Tsoy et al. (96)	Winblad (45)	MoCA	–	Adapted variant D WMS	Letter cancellation together with the Bourdon Correction Test	–	–	–	Cube, pyramid, truncated pyramid	–
Vlachos et al. (97)	Petersen (3)	CDR MMSE	A Greek multiple choice vocabulary test	GVLT, immediate and delayed recall MCG Complex Figure Test, immediate and delayed recall	TMT part A	TMT part B VF ASR Graphical Sequence Test MP months forwards and backwards	Semantic and phonological VF BDAE (BNT short form, and selected items from CIM)		BJLOT abbreviated form MCG Complex Figure Test copy condition CDT	TMT part A
Welstead et al. (98)	Albert (1)	MMSE	Matrix Reasoning	WMS-III LM	Symbol Search Digit Symbol Coding	Letter-number sequencing			WAIS-III BD	
Wu et al. (99)	–	MMSE	–	–	–	–	–	–	–	–
Xu et al. (100)	Petersen (7)	MMSE MoCA WMS-R	–	–	–	WAIS TMT	Rapid Verbal Retrieve	–	–	–
Yang et al. (101)	Petersen (3)	MoCA ADL CDR	–	–	–	–	–	–	–	–

a The abbreviations that are present in this table are reported in the Appendix.

**Figure 5:**
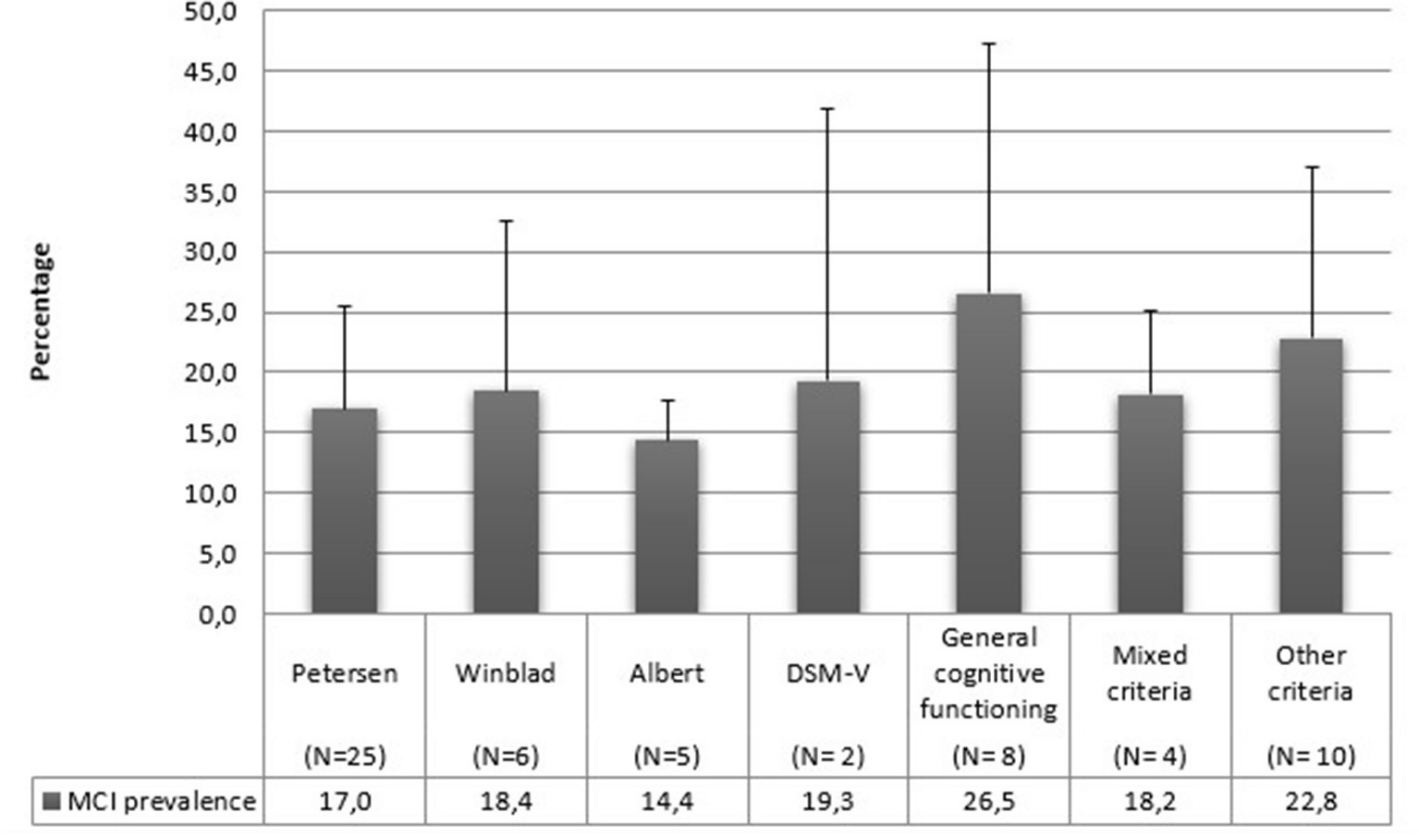
MCI mean prevalence rates according to diagnostic criteria.

Concerning diagnostic criteria, half of the studies referred to Petersen's criteria. All the utilized diagnostic criteria are reported in detail in Table 5 and Figure 5.

**Table 5:** Diagnostic criteria^a^.

**References**	**Diagnostic criteria**	**Global functioning**	**Subjective cognitive complaints**	**Cognitive decline**	**Objective cognitive impairment**	**Normal functional abilities**	**Absence of dementia**	**Normal mental status**
Afgin et al. (29)	Morris (30)	CDR = 0.5						
Alkhunizan et al. (31)	Trzepacz (32)	MoCA < 26						
Amer et al. (33)	MoCA < 26	MoCA < 26						
Anstey et al. (34)	Petersen (7)	–	–	–	–	–	–	–
Busse et al. (35)	Petersen (7)		√		√	√	√	
Busse et al. (35)	aMCI-modified: no criterion regarding subjective cognitive complaints				√	√	√	
Busse et al. (36)	Petersen (7)		√		√	√	√	
Busse et al. (36)	aMCI-modified: no criterion regarding subjective cognitive complaints				√	√	√	
Chuang et al. (37)	Albert (1)		√		√	√	√	
Clark et al. (38)	Jak (13) Criterion 1: At least one test blow the cut-off Criterion 2: At least two tests below the cut-off for one cognitive domain	-	-	-	-	-	-	-
Dimitrov et al. (39)	Petersen's criteria modified by Portet (40)		√	√	√	√	√	
Ding et al. (41)	Petersen (3)		√		√	√	√	
Dlugaj et al. (42)	MCI- original criteria (3, 45)		√		√	√	√	
Dlugaj et al. (42)	MCI- modified criteria: no criterion regarding subjective cognitive complaints				√	√	√	
Fernández - Blázquez et al. (43)	Albert (1)	CDR = 0.5						
Ganguli et al. (44)	Criterion 1	CDR = 0.5						
Ganguli et al. (44)	Criterion 2: *Ad hoc*							
Ganguli et al. (44)	Criterion 3: Petersen (3)		√		√	√	√	MMSE ≥ 21
Ganguli et al. (44)	Criterion 4: Winblad (45)		√		√	√	√	MMSE ≥ 21
Gavrila et al. (46)	Caracciolo (47)				√	√		MMSE ≥ 1DS
GjØra et al. (48)	DSM-5	-	-	-	-	-	-	-
González et al. (49)	Albert (1)		√	√	√	√		
Han et al. (50)	Petersen (3)		√		√	√		
Hanninen et al. (51)	Petersen (7)	CDR = 0.5	√		√	√	√	√
Jia et al. (17)	Petersen (3) Winblad (45)	CDR = 0.5			√	√	√	
Juarez-Cedillo et al. (102)	Petersen (95)		√		√	√	√	MMSE > 23
Juncos-Rabadan et al. (52)	Petersen (3)	–	–	–	–	–	–	–
Katz et al. (53)	Artero (54)		√		√	√	√	
Khedr et al. (55)	–	CDR = 0.5		√	√		√	
Kim et al. (56)	Petersen (3)	–	–	–	–	–	–	–
Kochan et al. (57)	Petersen (3)	–	–	–	–	–	–	–
Kumar et al. (58)	Petersen (7)	–	–	–	–	–	–	–
Lara et al. (59)	Albert (1)		√		√	√	√	
Lee et al. (60)	Petersen (3)		√		√		√	MMSE >1.5 DS
Li et al. (61)	Petersen (3)		√		√	√	√	MMSE > 23
Limongi et al. (62)	Dlugaj (42)				√	√	√	MMSE > 23.8
Lindgren et al. (63)	Knopman (64)	TICS-m = 28–31						
Liu et al. (65)	Albert (1)			√	√	√		
Liu et al. (66)	Albert (1)		√		√	√	√	
Lopez et al. (67)	–			√	√	√	√	
Lopez-Anton et al. (68)	Petersen (7)		√	√	√	√	√	√
Lopez-Anton et al. (68)	DSM-5		√	√	√	√		
Luck et al. (69)	Original criteria (45)		√	√	√	√	√	
Luck et al. (69)	Modified criteria: no criterion regarding subjective cognitive complaints			√	√	√	√	
Ma et al. (70)	Petersen (3)		√		√	√	√	√
Meguro et al. (71)	Petersen (7)		√		√	√	√	√
Mohan et al. (72)	Portet (40)		√	√	√	√	√	
Mooi and Hamid (73)	Shahar (74)	MMSE ≤ 21						
Moretti et al. (75)	Winblad (45) Definition A: no criterion regarding subjective cognitive complaints	–	–	–	–	–	–	–
Moretti et al. (75)	Winblad (45) Definition B: original criteria	–	–	–	–	–	–	–
Noguchi-Shinohara et al. (76)	Winblad (45)			√	√	√	√	
Ogunniyi et al. (77)	Petersen (9)	–	–	–	–	–	–	–
Paddick et al. (78)	Winblad (45)	–	–	–	–	–	–	–
Parlevliet et al. (79)	Krabbe (80)		√		√		√	
Peltz et al. (81)	Petersen (82)				√	√		MMSE ≥ 24
Petersen et al. (83)	Petersen (3)		√		√	√	√	
Radford et al. (84)	Winblad (45)	–	–	–	–	–	–	–
Rao et al. (85)	Petersen (3)		√		√	√	√	
Rentería et al. (86)	Jak (13)				√			
Robertson et al. (27)	Trittschuh (87)	–	–	–	–	–	–	–
Ruan et al. (88)	–	RCS = 6–7						
Sasaki et al. (89)	–		√		√	√	√	
Shahnawaz et al. (90)	Winblad (45)		√		√	√	√	
Shimada et al. (19)	Jungwirth (91)		√		√	√	√	
Sosa et al. (28)	Petersen (9)		√		√	√	√	
Su et al. (92)	Petersen (3)		√		√	√	√	√
Teh et al. (26)	Winblad (9)		√		√	√	√	
Tognoni et al. (93)	Petersen (7)		√	√	√	√	√	√
Tsolaki et al. (94)	Method 1: *Ad hoc* Method 2: Same criteria as method 1 but MMSE score is adjusted for age and schooling Method 3: Petersen (95) Method 4: Same criteria as method 1 but MMSE score is adjusted for age and schooling	MMSE/HINDI score						√
Tsoy et al. (96)	Winblad (45)		√		√	√	√	
Vlachos et al. (97)	Petersen (3)		√		√	√		
Welstead et al. (98)	Albert (1)		√		√	√	√	MMSE ≥ 24
Wu et al. (99)	–	MMSE score						
Xu et al. (100)	Petersen (7)		√		√	√	√	√
Yang et al. (101)	Petersen (3)	CDR ≤ 0.5 MoCA ≤ 23			√	√	√	

a The abbreviations that are present in this table are reported in the Appendix.

### Cognitive domains

**Figure 6:**
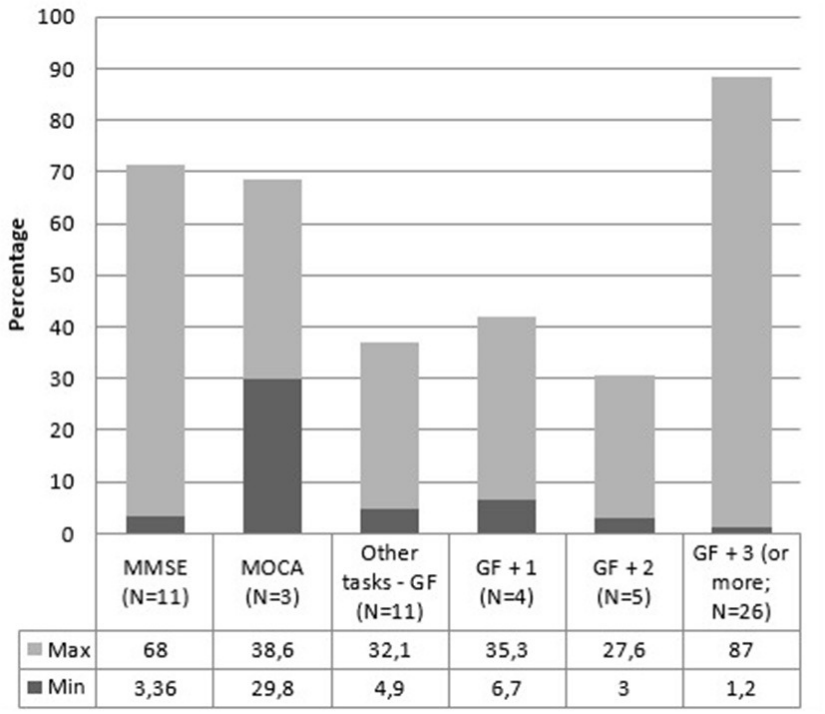
MCI prevalence range according to the number of assessed cognitive domains. MMSE, Mini-Mental State Examination; MoCA, Montreal Cognitive Assessment; GF, global functioning; GF + 1, global functioning + one domain; GF + 2, global functioning + two domains; GF + 3 (or more), global functioning + three domains (or more).

For what concerns cognitive domains, 29 articles out of 66 (44%) only measured global cognitive functioning, utilizing MMSE, MoCA, CDR, or other tasks such as SIDAM. In these studies, prevalence ranges from 3 to 68%. Five studies (7.6%) assessed one other domain (memory or executive functioning). The prevalence range in these articles goes from 6.7 to 35.3%. Six studies (9%) analyzed at least two cognitive domains, finding a prevalence that ranges from 3 to 27.6%. Lastly, 26 articles out of 66 (39.4%) evaluated at least three cognitive domains. The range goes from 1.2 up to 87% in these ones. Figure 6 and Table 6 report the MCI prevalence according to the cognitive domains assessed.

**Table 6:** Prevalence range according to the assessed cognitive domains^a^.

	**Articles**	**N. of articles**	**Prevalence range**
Global functioning	Afgin et al. (29) Alkhunizan et al. (31) Amer et al. (33) Fernández-Blázquez et al. (43) Kim et al. (56) Lindgren et al. (63) Liu et al. (65) Lopez-Anton et al. (68) Luck et al. (69) Ma et al. (70) Meguro et al. (71) Mohan et al. (72) Mooi and Hamid (73) Moretti et al. (75) Noguchi-Shinohara, et al. (76) Ogunniyi et al. (77) Paddick et al. (78) Parlevliet et al. (79) Radford et al. (84) Ruan et al. (88) Shimada et al. (19) Su et al. (92) Tsolaki et al. (94) Wu et al. (99) Yang et al. (101)	25	3–68%
Global functioning + one domain	Dimitrov et al. (39) GjØra et al. (48) Han et al. (50) Juncos Rabadan et al. (52)	4	6.7–35.3%
Global functioning + two domains	Clark et al. (38) Dlugaj et al. (42) Lee et al. (60) Lopez et al. (67) Xu et al. (100)	5	3–27.6%
Global functioning + three domains (or more)	Anstey et al. (34) Chuang et al. (37) Ding et al. (41) Ganguli et al. (44) González et al. (49) Hanninen et al. (51) Jia et al. (17) Juarez Cedillo et al. (102) Katz et al. (53) Kochan et al. (57) Kumar et al. (58)	26	1.2–87%
	Lara et al. (59) Li et al. (61) Limongi et al. (62) Liu et al. (66) Peltz et al. (81) Petersen et al. (83) Rao et al. (85) Rentería et al. (86) Robertson et al. (27) Sasaki et al. (89) Shahnawaz et al. (90) Teh et al. (26) Tsoy et al. (96) Vlachos et al. (97) Welstead et al. (98)		

a Articles that only evaluated aMCI prevalence have not included.

### Cut-off scores

**Figure 7:**
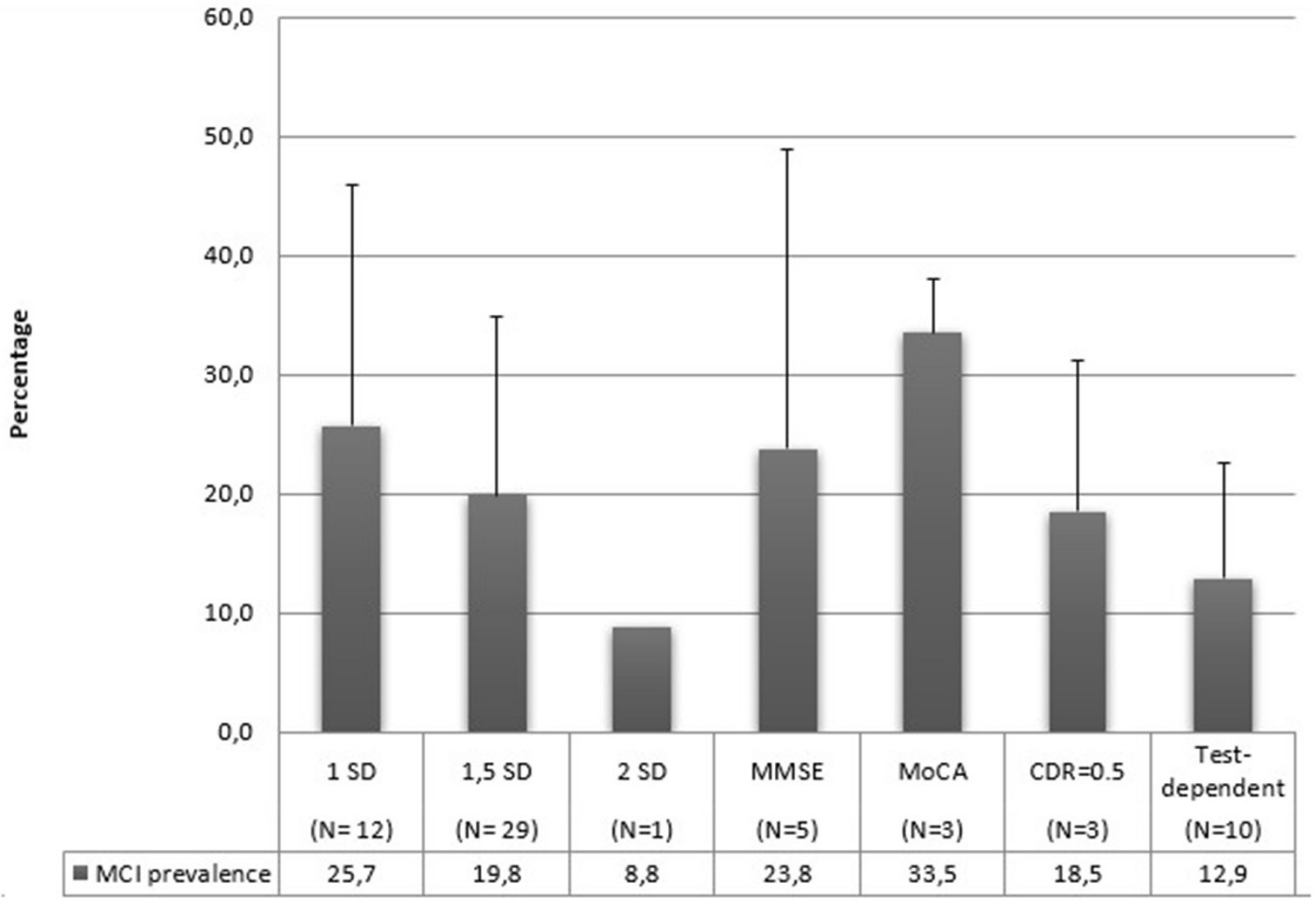
MCI mean prevalence rates according to cut-off scores.

In Figure 7, mean prevalence MCI rates are reported according to the utilization of different cut-off scores for the MCI diagnosis. Three studies (27, 57, 89) have utilized multiple scores for their diagnosis. MoCA scores refer to the normative score of < 26 to diagnose MCI. MMSE cut-off scores vary between 17 and 28. Test-dependent cut-off scores refer to specific values related to the assessment tools, for example a score of 6 or 7 points to the RCS was utilized by Ruan et al. (88) to diagnose MCI. Also in this case, standard deviations are very high, and the limited and different number of studies using a single criterium prevent us from any conclusion about their relationship with MCI prevalence.

## Discussion

This systematic review aimed to evaluate and compare the estimated prevalence of MCI. Ten years ago, one study (103) had already tried to compare these prevalence rates, highlighting a great variability ranging from 3% (104) up to 42% (54). The authors' purpose was to identify the sources of variation in MCI prevalence rates, but they encountered many challenges while trying to investigate this topic. Although many studies have been included since 2012, we faced similar difficulties in our systematic review, where we found an even greater range compared to the previous one reported by Ward et al. (103). Indeed, the selected studies of this review rate go from 1.2% (26) to 87% (27). The greater range of MCI prevalence observed in this review compared to that of Ward et al. (103) may have been due to an exponential increase in MCI studies over the past decade.

However, it is unclear whether these great discrepancies are caused by methodological problems, such as heterogeneity in the sample characteristics (size, age, gender, schooling, and nationality), assessment tools or procedures, or limits due to the operationalization of the MCI criteria. According to this last aspect, since its first proposal (7), MCI criteria have been evolving: at first, it was used only to describe impairments in the memory domain (7), while afterward, it broadened to embrace many other domains (3). This factor has contributed to a certain confusion regarding the MCI diagnostic methodology.

We can state that the differences in the diagnostic criteria have determined the biggest problems incomparing studies in our review. This aspect is highly complicated since many studies apply the same diagnostic criteria but operationalize them differently. For example, Chuang et al. (37) utilize Albert's criteria with a cut-off of 1.5 SD, while González et al. (49) utilize the same criteria with a cut-off of 1 SD. Using a 1.5 SD cut-off is more sensitive to catching cognitive decline than a 2 SD cut-off but necessarily less specific (20). The most utilized criteria in this review are those indicated by Petersen et al. (3, 7): indeed, half of the studies included in this review refer to these criteria. These operational differences can regard the use of different tasks to assess cognitive abilities, the assessment of different cognitive domains, or different cut-off values. It is a known fact that the operationalization of MCI criteria affects MCI prevalence estimates, and assessing MCI with different criteria, even if slightly modified, can lead to very different prevalence estimates. For example, Kochan et al. (57) modified their operationalizations, finding prevalence rates ranging from 4 to 70%.

Moreover, some authors have modified the original diagnostic criteria of MCI (1, 35, 36, 45), which has caused even greater heterogeneity. Indeed, there is often a criterion regarding subjective cognitive complaints (SCC) in the original criteria, but there is no consensus on its validity. Therefore, researchers often diagnose MCI in patients who do not report SCC using modified criteria. The elimination of this criterion results in higher prevalence rates: indeed, Busse et al. (36) utilized these modified criteria and found an increase from 8.5 to 16.8%.

The boundary between MCI and subjective cognitive impairment is complex, and it could seem arbitrary since it depends on an interaction between the properties of the employed tasks and the subject's characteristics (schooling, language, and cultural factors).

Moreover, the authors utilize different assessment tools for diagnosing MCI. In this review, 29 studies out of 66 only measured global functioning using MMSE, MoCA, CDR, or other tests. However, it is known that simpler, bedside screening tests like the MMSE and MoCA can be useful for screening purposes, but they may exhibit ceiling effects in those with the mildest levels of impairment (20).

As reported in Figure 6, the evaluation of global cognitive functioning alone or the evaluation of global functioning in addition to one, two, or more domains only highlights that evaluating at least three cognitive domains in addition to global functioning returns a higher prevalence. This result is quite predictable since evaluating more cognitive domains increases the possibility of identifying a compromised cognitive domain. However, it should be noted that the prevalence is lower when assessing global cognitive functioning plus two cognitive domains compared with assessing global cognitive functioning alone. Furthermore, in all cases, the range of MCI prevalence is very high. It is evident that the results are affected by other factors, such as the characteristics of the sample (e.g., age, gender, schooling) or the tests used to evaluate cognitive functions. Obtaining different prevalence using different tests can be obvious. For example, it is very different assessing attention with the Attentional Matrix test (105) rather than by using a more complex test that evaluates various aspects of attention, such as the Attentional Network Test (106) or a variant of it more suitable for evaluating attention systems in the elderly [e.g., (107, 108)]. Therefore, we can state that the prevalence of MCI is strongly affected by the choice of neuropsychological assessment parameters. This lack of a clear, standardized diagnostic methodology is the biggest cause of the highly heterogeneous prevalence rates found in this systematic review.

Besides these differences due to the MCI criteria, there are problems regarding the methodological aspects of the studies, that is, the sample characteristics (size, age, gender, schooling, and nationality) that we analyzed in the results section.

In particular, the minimum age for inclusion varied largely. Some studies included an older sample (≥60, ≥75, or ≥90), while others embraced much younger people (≥45, ≥50, or ≥55). This aspect could have influenced our prevalence estimates. The authors that include younger samples (≥45, ≥50 or ≥55; 26–33) found a prevalence range that goes from 3 to 34.4%. This range is lower than our whole range, implying that a lower prevalence could be caused by sampling younger people since cognitive deficits increase with aging. However, even among authors who include young adults in their sample, it is easily noticeable that there is great variability in MCI prevalence estimates (from 3 to 34.4%), and this aspect indicates that age is not the only variable that affects our results. However, there is no clear difference in the MCI prevalence in the different age groups. As already indicated, this result may depend on the age of the individual components that make up the group, the criteria and tests used for diagnosing MCI, and the transition from MCI to dementia in the older groups.

Gender composition is another factor that varies substantially in these studies, with a female share ranging from 43 to 75.9%. In literature, it is not known if gender has some effect on MCI prevalence. A meta-analysis by Au et al. (109) found a higher prevalence of naMCI in women. On the other hand, Petersen et al. (83) noted that MCI prevalence is higher in men. Some of our studies included a vast majority of women (≥70%; 34–38) and found a prevalence range that goes from 3 to 49%. This range is lower than our whole range, which could suggest that women present lower MCI prevalence than men, according to Petersen's findings (83). However, there is still great variability in this range, and it is not very easy to draw definitive conclusions.

Besides gender, another important factor to take into consideration is schooling. This aspect is very diverse in the retained studies, going from illiteracy to university education. Not having completed high school can increase the possibility of developing MCI (110). Therefore, it is possible that having included illiterate people in the sample has highly increased the resulting prevalence estimates. In our review, many studies included illiterate people in their sample (17, 28, 31, 33, 35, 36, 43, 46, 59, 65, 66, 73, 92). The prevalence range found by these studies goes from 6 to 68%, but these rates drop to 0.6% (28) in the articles that evaluate only aMCI. It is conceivable that MCI prevalence can increase by including illiterate people in the sample, but it is impossible to corroborate this hypothesis with the results from this review. Moreover, some studies did not specify the sample's schooling years, and this aspect complicates the interpretation of our results even further.

As concerns sample size, in this review, we compared studies with a few hundred participants with studies with a sample of a few thousand subjects, up to 15,000. 57.6% of our articles had a sample size larger than 1,000 participants. The sample size is an important variable to consider in prevalence studies. In general, sampling for prevalence surveys requires a precise evaluation, especially when the studied condition is very rare or has a tendency for geographical clustering. Therefore, the sample size estimate for prevalence studies is a function of expected prevalence and precision for a given level of confidence expressed by the z statistics (111). Most of the included studies did not specify the calculation method for their sampling, and 42.4% had a sample with < 1,000 participants. Moreover, three studies (31, 33, 78) had < 300 participants, and we considered this a risk of bias according to the guidelines (24). This discrepancy in sample sizes does not help make comparisons among studies.

Our data are also grouped by country. A recent review conducted by Pais et al. (112) has not found any difference in MCI prevalence based on the geographical region. On the other hand, another study considered eight countries (Cuba, Repubblica Dominicana, Perù, Messico, Venezuela, Porto Rico, Cina e India) and found prevalence estimates of aMCI ranged from 0.6 to 4.6% (28). MCI is a construct that is easily influenced by the dominant culture. In some countries, illiteracy is very high and, as stated above, education represents a protective factor against MCI (113). It is impossible to draw conclusions on this matter in our review since most of our articles are from Asian or European studies, and we do not have sufficient data on the other areas. However, it would be useful to conduct reviews for analyzing studies based only on one specific area since each country has a different educational system and illiteracy rates, which could result in different MCI prevalence rates. Also in this case, it is necessary to define whether the apparent absence of difference in prevalence can be real or represent a type I error due to the numerous methodological problems already mentioned, as is also suggested by the high standard deviations. Likewise, the use of different diagnostic criteria (Figure 5), the number of cognitive domains evaluated (Figure 6), and the used cut-offs (Figure 7) return a highly variable prevalence of MCI, attributable to the afore mentioned methodological problems.

It is easily noticeable how methodological aspects characterizing these studies are insufficient to account for the high heterogeneity that we found in our results. Therefore, we could conclude that the main factors contributing to our outcomes are various diagnostic and operational issues.

### Limits, implications, and suggestions for the future

This review highlighted several limitations in examining prevalence studies regarding Mild Cognitive Impairment. In particular, the main weakness is given by the great heterogeneity of the included studies. Indeed, these 66 studies have used different criteria and scales to evaluate cognitive decline and assessed different cognitive domains. Therefore, this makes it difficult to compare results across studies. Another critical point of the reviewed studies concerns the different characteristics of the sample (size, age, sex, schooling) that limit the possibility of making comparisons even more. Moreover, another possible limit is linked to the incomplete recovery of studies: selecting only studies published in English and Italian may have led to eliminating important articles, further limiting the generalizability of the results. Another main limitation of this systematic review concerns the exclusion of all studies published before 1999. This choice was made to focus only on the MCI diagnosis after Petersen's proposal (7). However, since the 1960's authors have been trying to find nosological categories to describe the presence of an isolated cognitive impairment in the elderly who are not affected by dementia [e.g., Benign Senescent Forgetfulness, Age Associated Memory Impairment, Age Associated Cognitive Decline, Cognitive Impairment No Dementia; (114)]. Therefore, our results could be strongly affected by these exclusion criteria. However, our main goal was to focus on MCI, and we chose these criteria based on other authors who adopted our same strategy. Indeed, Vanacore et al. (114) state that, by searching the term “MCI” on PubMed, there was an increase of 929% from 1999 to 2016.

Regardless of these limitations, at the end of this review emerges an effective need to develop a diagnostic system shared and standardized. Indeed, in the light of the results of this review, it would be advisable for future research to define a standardized and homogenous diagnostic system so that it will be possible to compare results across studies and ensure greater efficiency and accuracy in the diagnosis of MCI. In addition, it will be necessary to apply universal cut-off values, which have considered the different factors associated with cognitive decline, such as age, schooling, and even gender. Moreover, we need to develop a broad test protocol to assess precisely all the cognitive domains involved in MCI. Specifically, there should be a consensus on the number and the type of cognitive domains to evaluate, the cut-off scores to utilize, and the assessment tools to implement. It would also be useful to analyze the interactions between various cognitive systems and the implications of the two cerebral hemispheres, which vary with age (107) and may also present specific characteristics in MCI. It could also be interesting to analyze the association of these cognitive systems with the autonomous nervous system since they operate in close association (115, 116), and this could give us an additional biomarker for diagnosing MCI.

### Conclusions

Overall, this systematic review highlights a great variability in the prevalence of mild cognitive impairment. It is impossible to establish whether this heterogeneity is due to the adoption of different diagnostic criteria or dissimilarities in the sample's characteristics since all these factors are deeply interrelated. For example, some authors adopt the same diagnostic criteria (such as Petersen's) but use different assessment tools and cut-off scores. Therefore, we tried to stress this conclusion even further. We believe these results are caused by the lack of a standardized diagnostic methodology and an incomplete neuropsychological assessment. Consequently, many difficulties in this sector regard the effective implementation of interventions and therapies and the reliability of the results in the research field. Developing criteria and operationalizations that are more precise and standardized could allow improving benefits for all, firstly patients. Moreover, there is a strong need for a comprehensive standardized neuropsychological evaluation to allow a clear delineation of the aging profile associated with mild cognitive impairment. An evaluation of this type could be inserted into pathological aging prevention programs, and it could be useful for monitoring the progression from mild cognitive impairment to dementia.

In conclusion, the most relevant results of this review are two. First, the need to provide more methodological details in published studies on the composition of the sample in terms of age (e.g., reporting not only the mean age but also the age range and age distribution within the group), gender, schooling (also in this case reporting the distribution). Second, there is an urgent need to define a standard protocol for assessing cognitive decline, specifying which tests should be used, assessing all the main cognitive domains, and clearly defining the diagnostic criteria. In addition, the need to control the extent to which cognitive decline may represent the effect of other psychological (e.g., depression and anxiety) or behavioral (e.g., sleep quality) factors may be suggested, considering the close interconnection between these factors (107) and the effects of poor sleep on cognitive decline (117).

## Data availability statement

The original contributions presented in the study are included in the article/supplementary material, further inquiries can be directed to the corresponding author.

## Author contributions

MC had the idea of the work and made substantial contributions to its conception. FA, GF, and FF revised it thoroughly in each phase of the research. GM and AG acquired, analyzed, and interpreted the data. All authors contributed to the article and approved the submitted version.

## Conflict of interest

The authors declare that the research was conducted in the absence of any commercial or financial relationships that could be construed as a potential conflict of interest.

## Publisher's note

All claims expressed in this article are solely those of the authors and do not necessarily represent those of their affiliated organizations, or those of the publisher, the editors and the reviewers. Any product that may be evaluated in this article, or claim that may be made by its manufacturer, is not guaranteed or endorsed by the publisher.

## Appendix

3MS, Modified Mini-Mental State; ACE, Addenbrooke's Cognitive Examination tool; ADAS, Alzheimer's Disease Assessment Scale; ASR, Anomalous Sentence Repetition; BCST, Brookdale Cognitive Screening Test; BD, Block Design; BDAE, Boston Diagnostic Aphasia Examination; BIMC, Blessed Information–Memory-Concentration test; BJLOT, Benton Judgment of Line Orientation Test; BNT, Boston Naming Test; B-SEVLT, Brief-Spanish English Verbal Learning Test; BSRT, Buschke Selective Reminding Test; BVMT, Brief Visuospatial Memory Test; BVRT, Benton Visual Retention Test recognition; CAMCOG, Cambridge Cognitive Assessment-Revised; CAMDEX, Cambridge Examination for Mental Disorders of the Elderly; CASI, Cognitive Abilities Screening Instrument; CCD, Cross-Cultural Dementia; CCR, Category Cued Recall test; CDR, Clinical Dementia Rating Scale; CDT, Clock Drawing Test; CERAD, Consortium to Establish a Registry for Alzheimer's Disease; CERAD-K-C, Korean version of the CERAD Clinical Assessment Battery; CERAD-K-N, Korean version of the CERAD Neuropsychological Assessment Battery; CIM, Complex Ideational Material; CSI “D”, Community Screening Instrument for Dementia; CVLT, California Verbal Learning Test; DSB, Digit Span Backward; DSF, Digit Span Forward; DSST, Digit symbol substitution test; DST, Digit Span Test; eCog12, Everyday Cognition-12; FAB, Frontal Assessment Battery; FAS, FAS Verbal Fluency Test; FCSRT, Free and Cued Selective Reminding Test; FOME, Fuld Object-Memory Evaluation; GAI, Geriatric Anxiety Inventory; GDS, Global Deterioration Scale; GVLT, Greek Verbal Learning Test; HINDI, Hindi Mental State Examination; IDEA, Intervention for Dementia in Elderly Africans; IQCODE, Informant Questionnaire on Cognitive Decline in the Elderly; IU Token Test, Indiana University Token Test; KICA, Kimberley Indigenous Cognitive Assessment; LM, Logical Memory; -M, Modified; MCG, Medical College of Georgia; MDB, Mental Deterioration Battery; MES, Memory and Executive Screening test; MIS, Memory Impairment Screen; MMSE, Mini-Mental State Examination; MMSE-KC, Korean version of Mini-Mental State Examination; MoCA, Montreal Cognitive Assessment; MP, Motor Programming; NART, National adult reading test; NCGGFAT, National Center for Geriatrics and Gerontology-Functional Assessment Tool; NTBV, Neuropsychological Test Battery Vienna; NCT, Number Connection Test; NIHTB PVT, NIH Toolbox Picture Vocabulary Test; -R, Revised; RAVLT, Rey Auditory Verbal Learning Test/ AVLT, Auditory Verbal Learning Test; RBMT, Rivermead Behavioural Memory Test; RCS, Rapid Cognitive Screen; ROCF, Rey–Osterrieth complex figure test; RUDAS, Rowland Universal Dementia Assessment Scale; SBT, Short Blessed Test; SDMT, Symbol Digit Modalities Test; SIDAM, A Structured Interview for the diagnosis of dementia of the Alzheimer type, Multi-infarct dementia and dementias of other etiology according to ICD-10 and DSM-III-R; SIS, Six-Item Screener; SKT, Syndrom Kurztest; SRT, Story Recall Test; STMS, Short Test of Mental Status; TICS-m, Telephone Interview for Cognitive Status; TMT, Trail Making Test; VF, Verbal Fluency Test; WF, word fluency; VR, Visual Reproduction; WAB, Western Aphasia Battery; WAIS, Wechsler Adult Intelligence Scale; WHO-UCLA AVLT, World Health Organization- University of California–Los Angeles Auditory Verbal Learning Test; WLM, word list memory; WLR, word list recall; WMS, Wechsler Memory Scale.
